# Psychological status of medical security teams in Winter Olympic Games and Paralympics under COVID-19

**DOI:** 10.3389/fpubh.2024.1308573

**Published:** 2024-08-26

**Authors:** Xiaoyu Zhu, Yu Zhu, Zhiwei Qi, Ran Li, Yunlong Tan, Zhongwei Yang

**Affiliations:** ^1^Beijing Huilongguan Hospital, Peking University Huilongguan Clinical Medical School, Beijing, China; ^2^Peking University Third Hospital, Beijing, China

**Keywords:** anxiety, depression, emotional responses, mental health, public health emergencies, self-efficacy

## Abstract

**Background:**

Medical security work for the 2022 Beijing Winter Olympic and Paralympics faced enormous challenges under COVID-19. This study aimed to investigate the mental status of those medical team members to provide a reference for scientifically implementing medical security services for large-scale events.

**Methods:**

In this prospective cohort study, the Patient Health Questionnaire-9, Self-rating Anxiety Scale, General Self-Efficacy Scale, and Psychological Questionnaire for Emergent Events of Public Health (PQEEPH) were administered to 145 members of the medical team. A generalized mixed linear model was used to analyze the impact of work duration, position, on/off rotation, and gender on psychological status.

**Results:**

Work duration significantly impacted depression, anxiety, self-efficacy, and all dimensions of PQEEPH. Women scored higher than men in the PQEEPH dimensions of depression, neurasthenia, fear, and total score. Working status affected the dimensions of depression, neurasthenia, and total score. Deterioration in emotional state became apparent in the fourth week and recovered 1 week after the task concluded, while self-efficacy decreased from beginning to end.

**Conclusion:**

All the medical team members experienced emotional deterioration and decreased self-efficacy in medical security tasks. To maintain a medical team’s psychological wellbeing during large-scale activities, rotation times should be set reasonably, and adequate mental health services should be provided.

## 1 Introduction

The Olympic Games are a major traditional international sporting event. Hosting the Games can not only spread the Olympic spirit and promote international exchanges, but also demonstrates the host country’s soft power (1). However, the mass movement of people and large gatherings entail many challenges related to emergencies and public health issues (2). Furthermore, the potential risk of spreading infectious diseases could endanger the health system of the city, country, or region where the event takes place (3). The COVID-19 outbreak in December 2019 caused a global pandemic. The uneven vaccination rate, declining immunity, and emergence of new COVID-19 variants have brought new challenges to hosting major international sporting events (4). The pandemic caused the Tokyo 2020 Summer Olympic and Paralympic Games to be postponed for the first time ever. In November 2021, the World Health Organization reported about the new “Omicron” variant of COVID-19 (i.e., variant B.1.1.529) (5) that had enhanced transmissibility, raising concerns for public health amid mass gatherings, including sporting events. This presented greater challenges to large-scale crowd movements, and research shows that the pandemic is a hybrid threat comparable in scope to terrorism (6). The Olympic medical service team has also faced challenges associated with the pandemic (3). The 2016 Rio Olympics medical security team shouldered heavy responsibility under the Zika virus epidemic (7), and the COVID-19 pandemic has been even more complicated. In China, the COVID-19 pandemic has fundamentally changed people’s living conditions (8). Against such a grim backdrop, the 2022 Beijing Winter Olympics and Paralympics were held.

Even setting aside the challenges associated with the Olympic Games, in the case of infectious diseases, medical staff often face high-pressure and high-risk work situations and are more prone to various psychological problems (9). Working in isolation can also lead to negative psychological effects (10). Previous research during the Severe Acute Respiratory Syndrome (SARS) epidemic showed higher rates of mental health problems among medical staff (11–13). They were also more likely to experience traumatic experiences or symptoms of Post-Traumatic Stress Disorder (14) and had more severe symptoms of post-infection fatigue and anxiety (15). Multiple studies in China during the early stages of the 2020 COVID-19 pandemic revealed that medical staff, including non-frontline medical staff, exhibited significant vicarious traumatization and psychological problems (16, 17). Compared with non-clinical staff, front-line medical staff in the respiratory, emergency, infection, and intensive care units who had close contact with infected patients were twice as likely to experience anxiety and depression; and were 1.4 times more afraid of infection (18). A 2021 study conducted in Xinjiang, China, showed that under the normalized prevention and control policy, the prevalence of mental health problems among medical staff was as high as 20.25%, and nurses had a higher risk than doctors (19). In addition, studies have illustrated that the overall mental health of Chinese medical staff is poor (20).

Although the Beijing 2008 Olympic team provided medical security services that were acclaimed worldwide, they also had organizational and operational experience that provided a reference for the 2022 Winter Olympics and Paralympics (21). During a pandemic, the work of medical security is obviously more difficult. Providing good medical care in such situations requires a healthy medical team. The psychological health of medical staff is often undervalued compared with their physical health. This study aimed to explore the psychological status of the Beijing 2022 Winter Olympics medical team through evaluation indicators such as depression, anxiety, self-efficacy, and emotional responses to public health events, and to provide a reference for the deployment of medical services for large-scale events.

## 2 Materials and methods

### 2.1 Study design

This is a prospective cohort study.

### 2.2 Participants

In view of the ongoing COVID-19 pandemic, the International Olympic Committee announced on September 29, 2021, that all athletes and the medical team must remain inside the biosecurity bubble as part of the biosecurity protocol for the Olympic Games. This closed-loop management system included daily COVID-19 testing and restricted contact areas. Only travel to and from Olympic-related venues was allowed. The medical team was divided into two groups: an in-group and an out-group. The in-group was directly responsible for the medical security of Olympic-related personnel, whereas the out-group was responsible for rest in designated areas, handling paperwork, and emergency backup forces. Members of the medical team would take turns entering the in-group or out-group as needed, and have the opportunity to change group every 2 weeks.

On January 10, 2022, the medical security team gathered at the Chongli Winter Olympic Medical Center for the first assessment and then subsequently gathered every 14 days (window period ± 2 days). The final assessment was completed on March 22, 2022. All competitions ended on March 13, 2022, and the members of the medical team left after 2 weeks of centralized isolation and observation.

### 2.3 Assessments

General information, group conditions, and psychological status assessments of the medical team were collected using an online questionnaire. The first page of the questionnaire introduced the research, and all subjects signed an informed consent form online after reading it. This study strictly complied with the Declaration of Helsinki and was approved by the ethics committee of our research institution.

Depression was assessed using the Patient Health Questionnaire-9 (PHQ-9). PHQ-9 was formulated with reference to the diagnostic items of depression in the DSM-5. The questionnaire consists of nine items, each of which is scored from zero to three. The higher the score, the more severe the depressive symptoms. PHQ-9 has widely been used to assess depression, and Cronbach’s alpha was 0.86 (22).

Anxiety was assessed using the Self-rating Anxiety Scale (SAS). The SAS consists of 20 items scored on a scale of one to four, with higher scores indicating more severe anxiety symptoms. SAS has widely been used to assess anxiety, and Cronbach’s alpha was 0.93 (23).

Self-efficacy was assessed using the General Self-Efficacy Scale (GSES). The questionnaire consists of 10 items, and each item is scored from one to four. The higher the score, the higher one’s self-efficacy. Cronbach’s alpha was 0.91 (24).

Emotional responses to public health emergencies were assessed using the Psychological Questionnaire for Emergent Events of Public Health (PQEEPH). The PQEEPH consists of 25 items, which are grouped into five dimensions: depression, neurasthenia, fear, obsessive-compulsive anxiety, and hypochondria. Cronbach’s alpha was 0.692. This questionnaire can be used to assess the psychological responses of people over 16 years of age to public health emergencies. It was used in China during the SARS epidemic (25).

The study collected data by repeatedly measuring the same individual and utilized the self-control effect to control for bias.

### 2.4 Statistical analysis

All data analyses were performed using the IBM Statistical Product and Service Solutions (SPSS) data analysis software package (IBM Corp., Armonk, NY, United States, version 22.0). A generalized mixed linear model was used to evaluate the influence of various factors on the mental status of the medical staff. All tests were two-sided, and the significance level was set at 5%.

## 3 Results

### 3.1 Composition of the medical service team

The 2022 Winter Olympics medical service team had 145 members (43.4% male), including doctors (*n* = 49, 65.3% male), nurses (*n* = 43, 18.6% male), administration staff (*n* = 12, 66.7% male), technical (ECG, B-ultrasound, laboratory physician; *n* = 30, 36.7% male), and rear-service personnel positions (*n* = 11, 36.4% male).

### 3.2 Depression, anxiety, and self-efficacy

Work duration significantly impacted the depression, anxiety, and self-efficacy levels of medical staff, although position and work group had no significant impact on their anxiety and self-efficacy levels. In contrast, sex and work group had significant effects on depression (Table 1).

**Table 1:** The influence of various factors on PHQ-9, SAS, and GSES.

		Time	Sex	Position	Group
PHQ-9	F	9.825	5.003	1.088	6.249
	*p*	<0.001^*^	0.026^*^	0.361	0.013^*^
SAS	F	3.460	1.777	1.042	3.683
	*p*	0.004^*^	0.183	0.385	0.055
GSES	F	4.648	1.149	0.799	1.839
	*p*	<0.001^*^	0.284	0.526	0.176

^*^p < 0.05.

**Figure 1:**
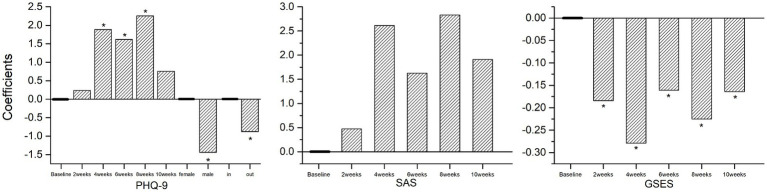
Coefficient of factors affecting PHQ-9, SAS, and GSES (^*^p < 0.05).

Depressed mood among medical staff increased significantly at weeks four, six, and eight compared to baseline. Men had lower depression scores than women, and members of the out-group had significantly lower depression scores than the in-group. Regarding anxiety scores, although there was an increasing trend from baseline for all participants from weeks 2 to 10, this was not statistically significant. Compared with the baseline, self-efficacy decreased significantly during the entire Winter Olympics support work period and failed to return to the baseline level 1 week after the mission ended (Figure 1).

### 3.3 Emotional responses to public health events

Work duration significantly affected all dimensions of the emotional responses of the medical staff to public health events. Women exhibited higher total scores than men in the dimensions of depression, neurasthenia, fear, and emotional response. The in-group scored higher than the out-group in the dimensions of depression, neurasthenia, and total score. Position had no significant impact on the scores for each dimension or total score (Table 2).

**Table 2:** The influence of various factors on PQEEPH.

		Time	Sex	Position	Group
Depression	F	7.750	5.820	0.742	6.143
	*p*	<0.001^*^	0.016^*^	0.563	0.013^*^
Neurasthenia	F	2.571	6.330	0.651	6.484
	*p*	0.026^*^	0.012^*^	0.626	0.011^*^
Fear	F	3.721	5.019	1.904	3.251
	*p*	0.003^*^	0.025^*^	0.108	0.072
Obsessive	F	4.062	1.675	0.735	3.382
	*p*	0.001^*^	0.196	0.568	0.066
Hypochondria	F	2.497	1.175	0.817	1.571
	*p*	0.030^*^	0.279	0.515	0.211
Total score	F	3.921	4.509	0.929	5.417
	*p*	0.002^*^	0.034^*^	0.447	0.020^*^

**Figure 2:**
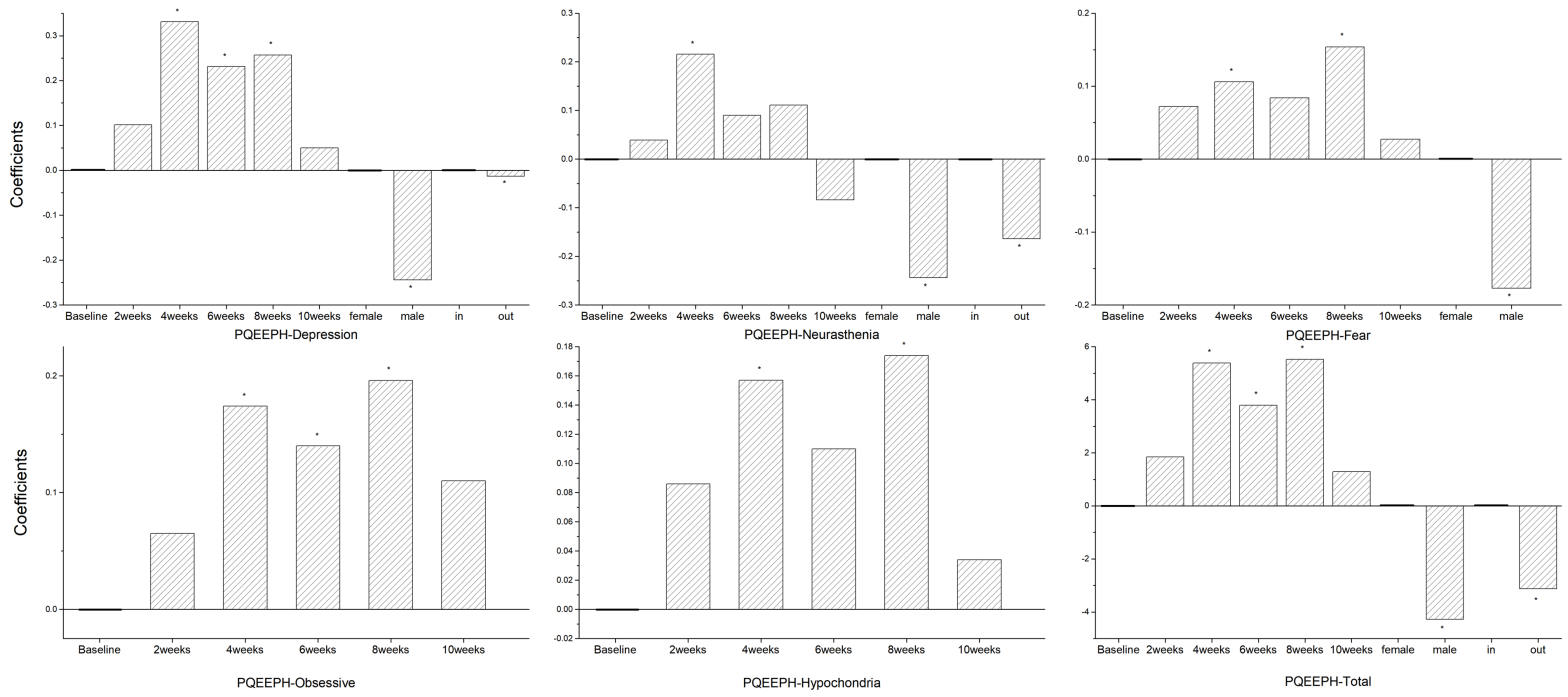
Coefficient of factors affecting PQEEPH (^*^p < 0.05).

The results for the depression dimension were consistent with those of the PHQ-9. The neurasthenia scores of the medical staff were significantly higher in the fourth week than the baseline, although changes in neurasthenia in men were milder than in women, and the neurasthenia score of the out-group was lower than that of the in-group. Compared with baseline, the medical staff showed significant fear and hypochondria at work weeks four and eight, with men having lower fear scores than women. At weeks four, six, and eight, the medical staff scored significantly higher than baseline on the obsessive-compulsive anxiety dimension. Overall, the total scores of emotional responses to public health events were significantly higher at weeks four, six, and eight than at baseline, with women and the in-group exhibiting more severe scores (Figure 2).

### 3.4 Statistical power

The statistical power of this study was calculated and found to be 0.868. This indicates a high probability that the study has correctly identified true effects and minimizes the risk of Type II errors.

## 4 Discussion

During an infectious disease epidemic, medical staff are more likely to come into contact with the source of the disease and face a higher risk of infection. Subhealthy and fatigued staff members may be more susceptible to the virus, leading to increased levels of anxiety related to uncertainty and fear of contagion (11, 26). Although medicine and medical management have undergone more than 10 years of development since the end of the original SARS epidemic, this is still not enough to relieve the psychological pressure on medical staff. A 2021 study conducted in Singapore showed that more than half of healthcare workers felt safer (54.8%), better equipped (72.1%), and better able to manage their workload (58.9%) during the current pandemic than during the SARS epidemic, and 77% believed that authorities and healthcare leaders were doing a better job now than they had before. However, less than half (47.3%) felt less stressed now compared to their past SARS experiences (27).

Unlike ordinary medical staff, the Winter Olympics medical team had to simultaneously deal with participating teams from different regions of the world. Medical conditions and equipment had more limitations, and medical care was performed in an isolated environment, creating more challenges.

Our study found that during the 10-week healthcare mission, healthcare workers in all positions, not just doctors and nurses but also administrative, technical, and support staff, experienced emotional deterioration and decreased self-efficacy.

During the service, the Winter Olympic medical team experienced persistent depression, increased anxiety, and emotional reactions such as neurasthenia, fear, obsessive-compulsive anxiety, and hypochondria. Most of the above changes did not recover after 2 weeks in the external group, suggesting that the deterioration of the mood of the medical staff was not entirely due to their current working status and infection risk, but might also be related to accumulated fatigue, limited range of activities, and pressure from future tasks. The final assessment occurred at the end of week 10, when the team completed all Olympic medical work, took a week off, and knew they would be able to return home in another week, yet their emotional status did not differ significantly from the baseline. Female members of medical teams experienced more severe symptoms of depression, fear, and neurasthenia.

In addition to emotions, self-efficacy is worth noting. Self-efficacy is a core concept in Bandura’s social cognitive theory (28). A good sense of self-efficacy can prompt individuals to cope with difficulties, while a poor working environment may destroy one’s sense of self-efficacy; this reduced self-efficacy will negatively impact the individual’s psychological status and work engagement (29, 30). We found that the self-efficacy of the medical team (regardless of position or sex) decreased significantly by the end of week two and had not yet recovered to baseline levels at the final assessment.

According to this study, the emotional status of most medical team members did not differ significantly from baseline at 2 weeks on the job, but significantly worsened after 4 weeks on the job. This deterioration persisted, indicating that 2–4 weeks is the time period that requires particular attention when deploying medical teams under public health pressure. The team can basically maintain its mental status within the first 2 weeks. If conditions permit, staff rotation for about 4 weeks may stabilize the emotional status and work efficiency of the medical team.

Regarding the effects of breaks, our research shows that taking either 2 weeks of rest when knowing they needed to keep on task or 1 week of rest after the task was completed did not reverse the deterioration in mental status associated with sustained high workload. Limitations in the range of movement during rest may have also decreased the effectiveness of rest. This suggests that longer rest or rotation schedules should be considered when deploying medical services for major events.

The strength of this study is that it focuses on the psychological wellbeing of medical teams at major events, an important but often neglected issue. The study offers a new perspective and provides valuable insights for medical deployment in such events.

However, there are some limitations in this study. The assessment of the medical team’s condition is restricted to scale-based evaluations. Including biological indicators would enhance the comprehensiveness of the assessment. Additionally, establishing the medical work group in its everyday state as a control group would enable a better comparison of the psychological states between large-scale event settings and routine medical work, highlighting their differences and influences in these contexts. From a research design perspective, this study primarily focused on observing the phenomenon of psychological deterioration among medical personnel. However, it did not incorporate intervention measures such as varying rest periods, relaxation training, or psychological interventions. As a result, the study missed the opportunity to identify effective methods for mitigating this deterioration. Furthermore, the study did not include an extended follow-up period to observe the complete recovery process of all indicators, thus failing to explore the recovery trajectory of the psychological state of medical personnel. Enhancing the research design in future studies could provide more practical and actionable guidance for managing similar scenarios in the future.

## 5 Conclusion

Medical team members at major events faced severe challenges during an infectious disease epidemic. These team members, especially women, experienced significantly deteriorated mental status with extended work duration. To address this situation, psychological support services should be considered for them. Less than 4 weeks of continuous work and more than 2 weeks of rest may be beneficial for them to maintain better wellbeing or to recover from poor wellbeing.

## Data Availability

The raw data supporting the conclusions of this article will be made available by the authors, without undue reservation.
